# Discovery of Novel MicroRNAs in Female Reproductive Tract Using Next Generation Sequencing

**DOI:** 10.1371/journal.pone.0009637

**Published:** 2010-03-10

**Authors:** Chad J. Creighton, Ashley L. Benham, Huifeng Zhu, Mahjabeen F. Khan, Jeffrey G. Reid, Ankur K. Nagaraja, Michael D. Fountain, Olivia Dziadek, Derek Han, Lang Ma, Jong Kim, Shannon M. Hawkins, Matthew L. Anderson, Martin M. Matzuk, Preethi H. Gunaratne

**Affiliations:** 1 The Dan L. Duncan Cancer Center, Baylor College of Medicine, Houston, Texas, United States of America; 2 Department of Medicine, Baylor College of Medicine, Houston, Texas, United States of America; 3 Department of Pathology, Baylor College of Medicine, Houston, Texas, United States of America; 4 Human Genome Sequencing Center, Baylor College of Medicine, Houston, Texas, United States of America; 5 Department of Molecular and Human Genetics, Baylor College of Medicine, Houston, Texas, United States of America; 6 Department of Molecular and Cellular Biology, Baylor College of Medicine, Houston, Texas, United States of America; 7 Department of Biology & Biochemistry, University of Houston, Houston, Texas, United States of America; 8 Department of Obstetrics and Gynecology, Baylor College of Medicine, Houston, Texas, United States of America; 9 The Translational Biology and Molecular Medicine Program, Baylor College of Medicine, Houston, Texas, United States of America; Cincinnati Children's Research Foundation, United States of America

## Abstract

MicroRNAs (miRNAs) are small non-coding RNAs that mediate post-transcriptional gene silencing. Over 700 human miRNAs have currently been identified, many of which are mutated or de-regulated in diseases. Here we report the identification of novel miRNAs through deep sequencing the small RNAome (<30 nt) of over 100 tissues or cell lines derived from human female reproductive organs in both normal and disease states. These specimens include ovarian epithelium and ovarian cancer, endometrium and endometriomas, and uterine myometrium and uterine smooth muscle tumors. Sequence reads not aligning with known miRNAs were each mapped to the genome to extract flanking sequences. These extended sequence regions were folded *in silico* to identify RNA hairpins. Sequences demonstrating the ability to form a stem loop structure with low minimum free energy (<−25 kcal) and predicted Drosha and Dicer cut sites yielding a mature miRNA sequence matching the actual sequence were considered putative novel miRNAs. Additional confidence was achieved when putative novel hairpins assembled a collection of sequences highly similar to the putative mature miRNA but with heterogeneous 3′-ends. A confirmed novel miRNA fulfilled these criteria and had its “star” sequence in our collection. We found 7 distinct confirmed novel miRNAs, and 51 additional novel miRNAs that represented highly confident predictions but without detectable star sequences. Our novel miRNAs were detectable in multiple samples, but expressed at low levels and not specific to any one tissue or cell type. To date, this study represents the largest set of samples analyzed together to identify novel miRNAs.

## Introduction

MicroRNAs (miRNAs) are short (∼22-nucleotide), single-stranded, non-coding RNAs that modulate gene expression. Through their binding to the 3′-UTR (untranslated region) of target mRNAs, miRNAs trigger either the degradation of the mRNA transcript or the inhibition of protein translation. miRNAs are initially transcribed as primary microRNAs (pri-miRNAs) and then undergo two processing steps. The first step is the generation, within the nucleus, of stem-loop precursors (pre-miRNAs ∼70 nt) by the enzyme Drosha. In the second step, the pre-miRNAs are exported into the cytoplasm and processed by the enzyme Dicer into a double stranded RNA duplex with two nucleotide 3′-overhangs, subsequently releasing the 17–25 nucleotide long mature miRNA. miRNAs are essential for normal mammalian development and help regulate genes and processes involved in cell growth, differentiation, and apoptosis [1]. Alterations in miRNA expression have been observed in a variety of human cancers [2], [3], [4] including ovarian cancer [5], [6], [7]. miRNAs also appear deregulated in other diseases affecting organs of the reproductive system such as uterine leiomyomas and endometriosis [8], [9], [10].

The number of miRNAs confidently identified in the human genome is currently over 700 (703 in miRBase v13.0). Though the actual number of miRNAs is not known, some *in silico* studies suggest as many as tens of thousands of miRNAs exist [11]. miRNAs have been traditionally discovered using experimental approaches such as cloning and Sanger sequencing [12]. However, the recent introduction of deep sequencing technology, enabling the simultaneous sequencing of up to millions of DNA or RNA molecules, has provided another option for the discovery of novel miRNAs that may have eluded previous efforts [13]. Previous studies using computational methods combined with high throughput experimental data—such as deep sequencing or tiling expression arrays—have successfully identified novel miRNAs [14], [15], [16], [17].

To date, we have exhaustively sequenced the small RNAome of over 100 human samples derived from various organs of the female reproductive system in both diseased and normal states, including ovarian samples (both normal epithelium and ovarian cancer), endometrial samples (from both healthy non-endometriosis and endometriosis patients), and uterine samples (both normal myometrium and benign and malignant uterine tumors). Studies on the functional roles of known miRNAs in the diseased states of these various systems are currently ongoing and either have been [18], [19] or will be described in other papers. However, the exceptional volume of sequence data generated from this work provided us a unique opportunity to mine for novel miRNAs that have eluded previous cloning and standard sequencing efforts. In the present study, we focused on novel miRNA discovery and have confidently identified both mature and star sequences for 7 previously unknown miRNAs using our deep sequencing data. We have also identified nearly 100 additional putative novel miRNAs with mid-to-high confidence which await additional confirmation.

## Results

Using Next Generation Sequencing, we dissected the small RNAome of 103 human specimens obtained or derived from various tissues from female reproductive system-related organs. Samples profiled are listed in Table 1. These specimens included short-term cultures of normal ovarian surface epithelium (NOSE), primary ovarian cancers and established cell lines (including serous, clear cell, and endometrioid histotypes), endometrium from normal and endometriosis patients, cyst wall from endometriomas, and uterine myometrium and uterine smooth muscle tumors (leiomyomas and leiomyosarcomas subtypes). Our sequencing efforts uncovered a large number of small RNA sequences that were unlike any known human miRNAs. We hypothesized that some of these unique sequences were novel human miRNAs and further examined them using a computational method for miRNA discovery that was developed by our group to integrate several published criteria for miRNA prediction [13], [20], [21].

**Table 1:** Reproductive tissues profiled.

Description	Number of samples (103 total)
Normal ovarian surface epithelium (NOSE), cell culture	4
Primary ovarian cancer, serous, malignant	8
Primary cancer, ovarian, serous, borderline malignant	1
Primary ovarian cancer, endometrioid, malignant	4
Ovarian cancer cell culture, clear cell	12
Ovarian cancer cell culture, serous	4
Endometrioma	10
Endometrium, non-endometriosis	10
Endometrium, endometriosis	3
Uterine leiomyoma	18
Uterine myometrium	18
Uterine leiomyosarcoma	9
Uterine leiomyosarcoma, cell culture	2

**Figure 1:**
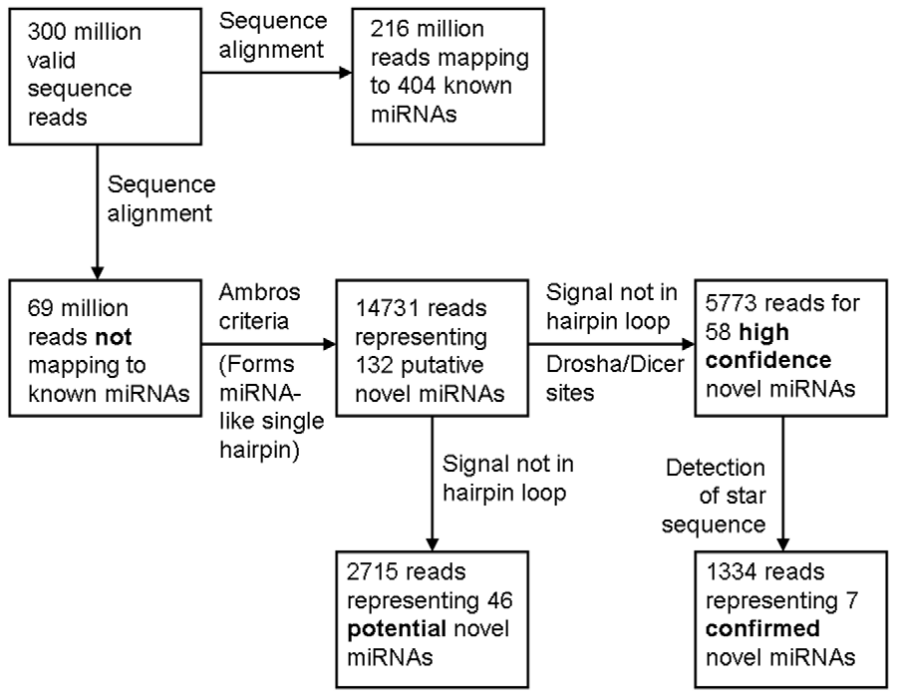
Flow chart of the mapping of deep sequence reads to novel miRNAs.

A schematic for our sequence analysis workflow is shown in Figure 1. In all, there were over 300 million valid sequence reads obtained from the 103 samples (“valid” sequence reads passing our quality control filters described in Methods), of which about 216 million mapped to known mature miRNAs, and an additional 15.6 million mapped to a known miRNA hairpin sequence (these sequences presumably representing the byproducts of miRNA processing). The remaining ∼69 million sequence reads not aligning with a known miRNA precursor (representing potential novel miRNAs) were mapped to the entire human genome. Those reads that mapped exactly to the genome were used to extract 200bp of genomic sequence flanking either side and folded as RNA using the Vienna RNA secondary structure prediction and comparison package [22]. The resulting putative novel hairpins were then filtered for single stem loop hairpins with the putative mature miRNA read sequence mapping to one side of the stem and satisfying the Ambros criteria for hairpin structures [20]. Valid novel miRNA hairpins whose putative mature miRNA sequence was identified in at least one of our samples were considered to represent *putative* novel microRNAs. In all, we identified 14,731 sequence reads representing 132 distinct putative novel miRNAs by virtue of their ability to form miRNA-like single hairpins. Here, we used the naming convention “hsa-bcm-miR-X” for each novel miRNAs, where “bcm” symbolizes “Baylor College of Medicine” and “X” is a unique identifier. Of the 132 novel miRNA, 20 mapped to multiple genomic loci; in this case, we used the mirBase convention of “-1,-2,-3, etc.” endings to the miRNA root name (e.g. “hsa-bcm-miR-15-1”, “hsa-bcm-miR-15-2”, and “hsa-bcm-miR-15-3”).

In addition to a name referring to the miRNA mature sequence, each novel miRNA genomic mapping received a unique hairpin identifier referring to the associated predicted hairpin structure. We used our putative novel hairpin sequences as precursors for alignment of all reads that did not map to a known miRNA precursor. In the next step of our analysis, we screened for putative novel hairpins that captured a collection of sequences (strong signal) mapping to a specific region of 15–25 nt within the reference hairpin on one side of the stem. Scattered sequence mappings across the full length of hairpin, a strong signal in a limited region that mapped to the loop, and hairpins mapping to known tRNA or ribosomal RNA regions were rejected. The strong signal was expected to contain short 17–25 nt sequences that exhibited a stable 5′-end and significant length heterogeneity at the 3′-end. The sequence with the highest copy number was considered the putative novel mature miRNA. The other related sequences were hypothesized to have been generated through ‘imperfect’ Dicer processing events reported by Morin *et al.* [23] and Reid *et al.* [24]. Additionally, predicted Drosha and Dicer cut sites must have been able to yield a mature miRNA sequence that matched to the actual miRNA sequence read. If a putative novel miRNA fulfilled all of the above, we denoted the novel miRNA as representing a “high confidence” prediction. In all, we identified 5,773 sequence reads representing 58 distinct *high confidence*, novel miRNAs.

For confirmation of these *high confidence* novel miRNAs, we needed to detect the star sequence in addition to the mature miRNA sequence in our sequence collection (though presumably at a much lower frequency since star sequences are degraded and usually occur at significantly lower levels). We defined the potential star sequence as the sequence base pairing to the potential mature sequence on the novel hairpin, correcting for the 2-nt 3′ overhangs which are known to be typical for Dicer processing [14], [23]. We denoted high-confidence novel miRNAs as “confirmed” if the star sequence was independently detected along with the mature sequence, as the novel miRNA sequence in question was then shown to fulfill all of the criteria that could be applied to known miRNAs. In all, we identified 1334 sequence reads representing seven distinct *confirmed* novel miRNAs.

**Figure 2:**
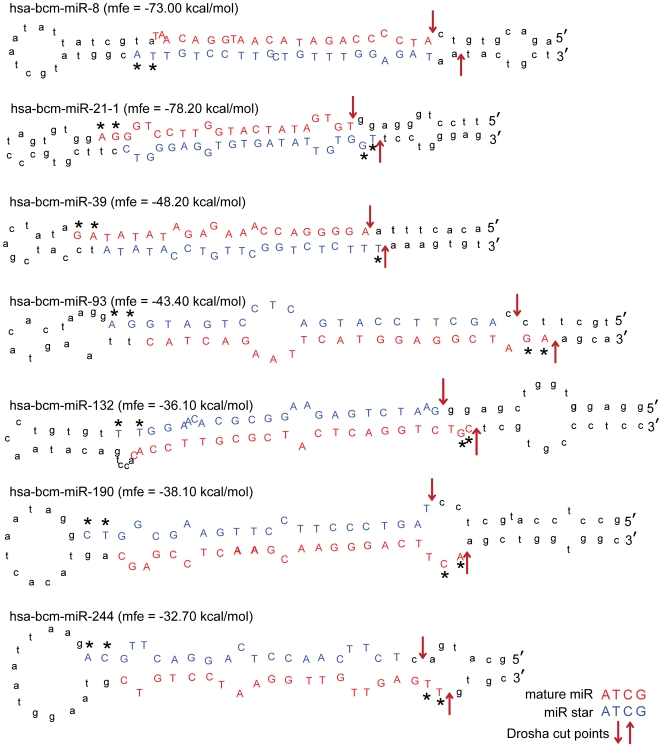
Predicted secondary structures of the precursor hairpins for the seven confirmed novel miRNAs. *Mfe*, predicted mean free energy level of the hairpin. Mature miRNA and miRNA star sequences—both detected by deep sequencing—are denoted (red and blue, respectively), as well as predicted Drosha cut points (red arrows). Asterisks indicate the small 3′ overhangs which are known to be typical for microRNAs.

The complete list of confirmed and/or high confidence novel miRNAs is provided in Table 2. The inability of our sequencing efforts to discover the star sequence for the 51 high confidence (but not “confirmed”) candidates does not necessarily eliminate them as representing novel miRNAs. Star sequences (passenger strands) are usually degraded after Dicer processing of the hairpin and therefore are present in much lower abundance than the active miRNA (guide strand). In particular, when the sequence copy numbers of a mature miRNA are relatively low, an even lower abundance of the miRNA star form may make it undetectable. In fact, for many of the low abundance known miRNAs that we identified (including hsa-miR-1301, hsa-miR-183, hsa-miR-203, and hsa-miR-371), we were unable to detect their representative star sequence (data not shown). For the seven novel miRNAs confirmed by virtue of star sequence identification, the predicted secondary structures of the precursor hairpins are represented in Figure 2. For most of these hairpins (with the possible exception of the hairpin formed by “hsa-bcm-miR-8”), the small 3′ overhangs, which are known to be typical for Dicer processing, were evident.

**Table 2:** Novel microRNAs identified.

bcm-miR	UCSC Blat	Sequence	Position (st = strand)	Samples detected
*Novel microRNAs, confirmed by detection of star sequence*				
8	Intergenic	ATCCCCAGATACAATGGACAAT	chr2:207682912–207683088 st−	12
21-1	Intron BC058547	TGTGATATCATGGTTCCTGGGA	chr9:136881792–136881939 st−	2
39	LINE	AGGGGACCAAAGAGATATATAG	chr6:120378000–120378122 st+	5
93	Intergenic	CATCAGAATTCATGGAGGCTAGA	chr3:48332858–48332955 st−	1
132	Intron OSBP2	CACCTTGCGCTACTCAGGTCTGC	chr22:29457541–29457630 st+	7
190	Intergenic	TAGTCCCTTCCTTGAAGCGGTC	chrX:149146896–149146977 st+	4
244	Intron EDA-A2	CTGTCCTAAGGTTGTTGAGTT	chrX:69159432–69159498 st+	16
*Novel microRNAs identified with high confidence, but no star sequence detected*				
3	Intergenic	ACAGGAAAGAATAAGAAGTCAT	chr17:20107032–20107214 st−	2
15-1	Intergenic	TGGGGCGGAGCTTCCGGAG	chr16:15156208–15156360 st−	6
15-2	Intergenic	TGGGGCGGAGCTTCCGGAG	chr16:2125979–2126131 st−	6
15-3	Intergenic	TGGGGCGGAGCTTCCGGAG	chr16:16311259–16311411 st+	6
19	Intron CRYGN	AGGTGCTCCAGGCTGGCTCACA	chr7:150761508–150761658 st−	1
21-2	Intron BC058547	TGTGATATCATGGTTCCTGGGA	chr9:136881157–136881228 st−	1
22	Intron PHC2	GATGAGGATGGATAGCAAGGAAG	chr1:33570591–33570735 st−	16
35	Intergenic	GAGCAATGTAGGTAGACTGTTT	chr12:122586909–122587034 st+	1
43	Intergenic	TGTCCTCTAGGGCCTGCAGTCT	chr22:34061633–34061751 st+	5
46	CDH2	CGGGGAGAGAACGCAGTGACGT	chr15:91248611–91248727 st+	10
48	Intergenic	AGCGCGGGCTGAGCGCTGCCAGTC	chr5:92982149–92982263 st−	1
53-1	ROR2/LINE	AAAGGCATAAAACCAAGACA	chr9:93438354–93438464 st+	14
53-2	LINE	AAAGGCATAAAACCAAGACA	chr9:93438367–93438448 st−	14
60	Intergenic	TGTGTGGATCCTGGAGGAGGCA	chr9:129492787–129492895 st−	2
70	Intergenic	TAACGCATAATATGGACATGT	chr5:170746265–170746369 st−	2
72	Intergenic	TTCTCAAGAGGGAGGCAATCAT	chrX:146139351–146139453 st−	4
75-1	Intergenic	TTTGGGACTGATCTTGATGTCT	chr12:68264769–68264870 st−	4
92-1	Intron WBSCR17 −	AAGGAACCAGAAAATGAGAAGT	chr7:70410594–70410692 st−	1
92-2	Intron WBSCR17 +	AAGGAACCAGAAAATGAGAAGT	chr7:70410596–70410690 st+	1
94	Intergenic	TGAGGGACAGATGCCAGAAGCA	chr2:69184307–69184404 st+	3
99	Intron DMD	TTGAGGAAAAGATGGTCTTATT	chrX:32511694–32511790 st−	1
108	Intergenic	CGGCGGGGACGGCGATTGGTC	chr11:61339205–61339300 st−	1
111	Intergenic	AGCTTTTGGGAATTCAGGTAG	chr4:153629927–153630020 st−	4
113	Intergenic	AAGAGGAAGAAATGGCTGGTTCTCAG	chr1:245431892–245431985 st−	1
118	Intergenic	AGGATTTCAGAAATACTGGTGT	chr11:126363560–126363652 st−	3
120	Intergenic	GCTCGGACTGAGCAGGTGGG	chr1:26105440–26105532 st−	7
122	Intergenic	ACAGGGCCGCAGATGGAGACT	chr6:159105681–159105773 st−	1
127	Intergenic	TTTGTATGGATATGTGTGTGTAT	chr8:78041555–78041645 st−	1
135	Intron SCHIP1	GCAGAGAACAAAGGACTCAGT	chr3:160483129–160483217 st+	2
142-1	Intron RP11-529I10.4 +	AAGGGCTTCCTCTCTGCAGGA	chr10:103351160–103351248 st+	23
142-2	Intron RP11-529I10.4 −	AAGGGCTTCCTCTCTGCAGGA	chr10:103351160–103351248 st−	23
150	SINE	GGCGACAAAACGAGACCCTGT	chr6:155216181–155216267 st+	7
158	Intergenic	TGAGGAGATCGTCGAGGTTGG	chr8:96154315–96154400 st−	1
159	Intron TRPC6	ACTGATTATCTTAACTCTCTGA	chr11:100895761–100895846 st−	1
160	LINE	TCTCTGAGTACCATATGCCTTGT	chr3:101165848–101165932 st−	1
164	Intergenic	AGAAGGGGTGAAATTTAAACGT	chr16:14902866–14902949 st+	4
167	Intergenic	TCTGGCCTTGACTTGACTCTTT	chr12:103509541–103509624 st+	1
171	LTR	AACTAGTAATGTTGGATTAGGG	chr3:79639727–79639809 st+	1
172	Intergenic	TGCCTGGAACATAGTAGGGACT	chr1:62317042–62317124 st−	8
189	Intergenic	AGATGTATGGAATCTGTATAT	chr14:27172246–27172327 st−	2
191	Intergenic	ATATGTATATGTGACTGCTACT	chr10:58734245–58734325 st−	1
192	Simple repeat	ATCATGTATGATACTGCAAACA	chr17:72597094–72597174 st+	3
203	Intergenic	ACAGTGAGGTAGAGGGAGTGC	chr4:9689335–9689412 st−	5
204	Intron AK094607	CAGGCAGTGACTGTTCAGACGTC	chr1:98283407–98283484 st−	17
210-1	Intron FASTKD2 +	GCTGCACCGGAGACTGGGTAA	chr2:207356202–207356278 st+	14
210-2	Intron FASTKD2 −	GCTGCACCGGAGACTGGGTAA	chr2:207356203–207356277 st−	14
212	Intergenic	AAGAGAACTGAAAGTGGAGCCT	chr6:36698191–36698267 st−	1
215	Intron COL5a2	GCAGTAGTGTAGAGATTGGTT	chr2:189706007–189706082 st−	9
219	Intergenic	TGTGTTAGAATAGGGGCAATAA	chr9:18563304–18563377 st+	1
223	Intergenic	TGAGGATATGGCAGGGAAG	chr2:134601163–134601236 st+	1
226-1	Intron LONRF1 −	TGGCCAAAAAGCAGGCAGAGA	chr8:12629112–12629184 st−	1
226-2	Intron LONRF1 +	TGGCCAAAAAGCAGGCAGAGA	chr8:12629117–12629179 st+	1
230	Intergenic	CAGGCGTCTGTCTACGTGGCTT	chr17:77032732–77032802 st−	1
232	LTR	CAGGTAGATATTTGATAGGCAT	chr9:111313576–111313646 st−	2
245	Intergenic	TTCGCGGGCGAAGGCAAAGTC	chr1:247087199–247087265 st+	5
261	Intron ACBD6	TAAATAGAGTAGGCAAAGGACA	chr1:178674081–178674139 st−	4
262	ncRNA	TGGGCTAAGGGAGATGATTGGGT	chrX:153650065–153650123 st+	26
265	Intergenic	GGAGGAACCTTGGAGCTTCGGC	chr22:29886048–29886105 st−	49
268	SINE	GAGGCTGATGTGAGTAGACCACT	chr18:31768049–31768103 st−	3

We also identified an additional 46 putative novel miRNAs (representing 2,715 sequence reads) that were flagged as “potential,” pending additional confirmation. These candidates had weak predicted Dicer and Drosha processing sites in the predicted hairpin structure, yet at the same time, these structures did show stable 5′ ends and variable 3′-ends. One of these potential candidates (“hsa-bcm-miR-49”) fulfilled all the criteria for the candidates at the high confidence level, and also mapped to a known snRNA. Since snoRNA ACA45 was found to be processed into a miRNA [25], hsa-bcm-miR-49 might represent another snRNA that can be processed into a miRNA. The complete set of confirmed, high confidence, and potential novel miRNA candidates, as well as the rest of the candidates out of the original list of 132 putative novel miRNAs that failed our additional stringency criteria, are provided as Supporting Data File S1. This file includes the information on the samples in which each miRNA was identified. The hairpin structure predictions for each of the 132 putative novel miRNAs is provided as Supporting Data File S2.

**Figure 3:**
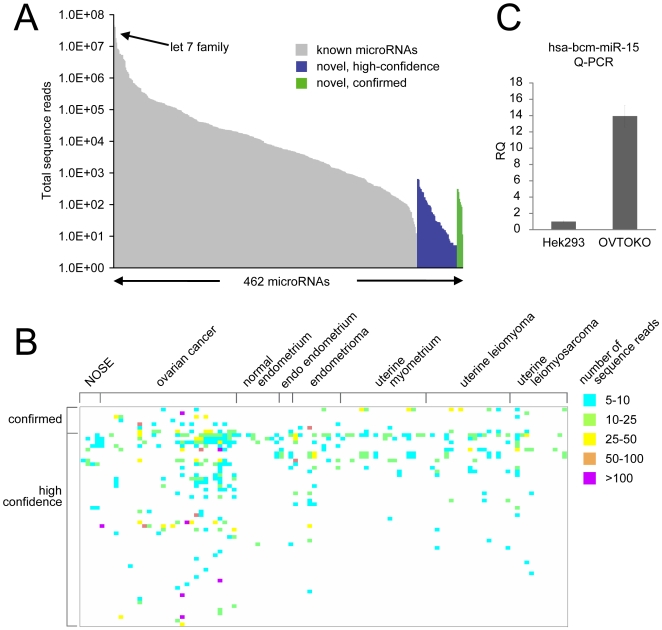
Relative abundance levels of novel miRNAs across samples. (**A**) Total number of sequence reads (summed across all samples) for both known miRNAs (404 in all) and novel miRNAs (note the log base 10 scale). *High confidence*, novel miRNA was found to form part of a miRNA-like hairpin, yet the hairpin star sequence was not detected by the deep sequencing (51 in all); *Confirmed*, novel miRNA formed part of a miRNA-like hairpin, with the star sequence also being detected (seven in all). (**B**) Heat map representing the number of sequence reads mapping to each novel miRNA hairpin across the samples. (**C**) Detection of hsa-bcm-miR-15 by qPCR. RQ, Relative Quantification (HEK293 sample as reference). hsa-bcm-miR-15 was detected at ∼300 sequence reads in OVTOKO cells and ∼100 reads in HEK293.

Figure 3A shows the relative abundance levels of both our confirmed and high confidence novel miRNAs across the samples profiled. When compared to the set of known miRNAs from our samples, our novel miRNAs were at much lower abundance levels. Even the most abundant novel miRNAs were at levels lower than most of the known miRNAs and several logs lower than what was observed for the let-7 family. Most of our confirmed and high confidence novel miRNAs were detected in multiple samples with various sequence copy numbers (Figure 3B as well as Table 2). Over 60% of the putative miRNAs were detected in more than a single sample. The novel miRNAs did not appear to be preferentially expressed in one reproductive tissue type versus another (Figure 3B). Many miRNAs were detectable in multiple reproductive tissue types. Statistically, we were not able to identify novel miRNAs as being specific to a diseased tissue as compared to the corresponding normal tissue type. Using an alternative method from deep sequencing, we were able to confirm expression by qPCR of one “high confidence” novel hsa-bcm-miR-15 (Figure 3C), which was the seventh most abundant novel miRNA in our list (and the most abundant miRNA detected in cell lines for which we had RNA).

## Discussion

The discovery of miRNAs has revealed a previously unanticipated layer of regulation that integrates the transcriptome (the complete set of RNAs expressed in a cell) with the proteome (complete set of proteins expressed). The ∼700 miRNAs uncovered from the human genome so far (as cataloged in miRBase) are predicted to target and repress over 60% of the protein coding genes [26]. Genome-wide miRNA predictions estimate that the true number of miRNAs may be anywhere from 10–100 times more than the current numbers [11]. Indeed, large-scale cloning and deep sequencing efforts in the last few years have led to a doubling of the number of human miRNAs from ∼470 (miRBase v8) to ∼700 (miRBase v13) and the miRNA targeted transcriptomes from >30% to >60% protein coding genes [26], [27], though the number of discovered miRNAs still falls short of the upper predicted limits of tens of thousands.

We used Next Generation Sequencing (NGS) technology to identify novel miRNAs in the female reproductive tract of women. In addition to our 7 confirmed novel miRNAs, many of the 51 high confidence novel miRNAs could also be eventually confirmed. The only feature that distinguishes a high confidence novel miRNA from a confirmed miRNA by our criteria is experimental evidence for both the miRNA and its star sequence within the collection of NGS sequences from a given samples. The novel miRNAs that we discovered occur generally in low abundance (<1000 copies in 2–10 million sequences per sequencing lane for a sample). It is therefore, highly likely that deeper sequencing of the tissues we sampled or other new tissues where they may occur in greater abundance may reveal a miRNA star sequence for the majority of high confidence miRNAs in which case they can be converted to confirmed status.

Previous deep sequencing efforts to identify novel miRNAs have typically relied upon data from a single human sample [14], [16], [17], [23] while one recent study used tiling array data from 14 human cell lines [15]. To date, this study represents the largest set of samples analyzed together by deep sequencing to identify novel miRNAs. The large collection of tissue and cell line samples used in this study allowed us to capture a large number of novel miRNA candidates detectable in some samples but not others. In fact, only one novel candidate (“hsa-bcm-miR-265”) was detected in more than a third of the samples, with about 40% of the 141 putative miRNAs being detected in only a single sample. Arguably, our approach has allowed us to uncover many more novel miRNAs than what might be uncovered in a single sample. For instance, the recent miRDeep study [14] uncovered 10 novel miRNA candidates in the HeLa cell line, while we uncovered 104 potential candidates (seven confirmed, 51 high confidence, and 46 potential) across all of our 103 samples. The novel miRNAs that we identified were typically low abundance as compared to most of the known miRNAs, explaining how they might have eluded previous efforts of detection.

Since sequencing is becoming faster, cheaper, and deeper, we anticipate the number of miRNAs to possibly approach the numbers estimated by some whole genome prediction algorithms. If this happens, it is possible that the miRNA-regulated transcriptomes include most of the genes in our genome, making them widespread agents of gene silencing. From the data that we have generated, it is clear that the most abundant miRNAs in mammalian systems have likely been found. It is possible that the lower abundance novel miRNAs uncovered here are present in higher abundance in as yet unanalyzed tissues or cell types. It is also possible that there are major miRNAs (i.e., the majority of known miRNAs) that have a strong influence on gene silencing and minor miRNAs (i.e., the majority of novel miRNAs) that have a subtle effect on fine tuning gene expression. In any event, only when the complete miRNAome is determined can we understand the full impact of miRNAs on gene expression on a genome-wide scale. Our novel miRNA detection study has added significantly to the work to complete the miRNAome.

## Methods

### Ethics Statement

Patients undergoing elective gynecologic surgery at Ben Taub General Hospital or St. Luke's Episcopal Hospital were approached prior to surgery for participation. After written informed consent, patients underwent scheduled surgical procedures. All tissues collected for this study were collected under Baylor College of Medicine Institutional Review Board (IRB) approval and IRB approval from each individual hospital.

### RNA Extraction and Small RNA Isolation

RNA was extracted from tissues or cell lines using the mirVana miRNA Isolation Kit (Ambion, Austin, TX) per manufacturer's instructions for total RNA isolation. RNA quality and the presence of small RNAs were inspected on a 2100 Bioanalyzer (Agilent). After strict RNA quality was assured, 15 µg of total RNA was used for small RNA library creation using Illumina's DGE small RNA sample prep kit per manufacturer's instructions. Purified cDNA was quantified with the Quant-iT PicoGreen dsDNA Kit (Invitrogen) and diluted to 3 pM for sequencing on the Illumina 1G Genome Analyzer (Solexa)(University of Houston). Each library was sequenced in a single lane.

### Small RNA Mapping

Sequence reads with Solexa 3′ adapter (the read length being 36 nt) were picked for miRNA mapping (the same adaptor being used for each sequencing run). Each sequence read was passed through a number of quality control filters. Reads which did not pass the Illumina chastity and no-calls filter were removed. Reads with copy number less than 4, length less than 10 nt, or more than 10 consecutive, repetitive nucleotides were removed. Reads matching the *E. coli* genome were removed using WU BLAST [28]. The remaining reads (which we termed “valid” sequence reads) were compared with known mature miRNA hairpins (miRBase 13.0) [29]. Reads were mapped as either exact match or loose match (loose match only for reads without an exact match). For loose match (which would account for miRNAs being subjected to non-templated nucleotide changes and RNA editing [24]), up to three mismatches were allowed in a single alignment (by our experience, allowing four mismatches yielded diminishing returns decreasing the cost benefit of engaging all sequence reads that pertain to a specific miRNA versus the increase in alignment times); for loose matches, we used a custom Smith-Waterman local alignment algorithm, where our gap penalty was −3, match score was 2, and mismatch penalty was −1, with a cutoff of 1.46. The reads that did not align to any known miRNA were passed to our novel miRNA discovery platform as described below.

### Novel miRNA Discovery

Our basic approach for novel miRNA discovery has been described previously [13]. Briefly, each sequence which passed our quality control filters was first mapped on the reference genome sequence (hg18) plus 200 bases of flanking the sequence on either side were extracted to find the putative hairpin. This extracted sequence was then folded using RNAfold of the Vienna RNA folding package [22], in order to determine secondary RNA structure. After folding of the long (∼425 nt) sequence (and confirmation that it contains a miRNA-like hairpin with the sequenced tag in the proper place) the putative precursor sequence was trimmed down to include only the hairpin bases (60–150 nt) and refolded to confirm that the structure is maintained in both the larger and shorter contexts. To determine if a structure forms a plausible miRNA hairpin, we applied the three Ambros criteria [20]: 1) the mature putative miRNA sequence must rest on one side of a single hairpin; 2) the putative miRNA sequence must bind relatively tightly (i.e. at least 16 bound bases in the first 22 or fewer bases of the miRNA) within the hairpin stem containing no large or energetically unfavorable loops (i.e. a single loop with more than 6 bases in the arm of the hairpin); and 3) the putative hairpin must have a miRNA-appropriate energy (free energy below −25 kcal/mol). We recognized small RNA sequences as representing putative novel miRNA if all the above were met.

We carefully curated the set of putative novel miRNAs and divided them into four different categories: “not likely,” “potential,” “high confidence,” and “confirmed.” Candidates were flagged as “not likely” if any of the following was determined: the mature sequence did not map clearly within a specific region of 15–25 nt of the predicted hairpin (e.g. were scattered evenly across the full length of the hairpin), or fell within the hairpin loop, or mapped to known tRNAs or rRNAs. (Though the minimum length of a miRNA is thought to be 17 nt, we used a lower limit of 15 for the mapping region, to account for the fact that microRNAs exhibit significant length heterogeneity at the 3′ end and to capture all isomiRNAs [23], [24].) Candidates were categorized as “high confidence” if they passed all of the above criteria, and in addition formed a hairpin with predicted Drosha and Dicer cut sites that were able to yield a mature miRNA sequence matching the actual miRNA sequence read, as well as not mapping to known snoRNAs or snRNAs. Candidates were categorized as “potential” if they had weak predicted processing sites in the hairpin, yet the hairpin showed a stable 5′ end (candidates representing snoRNAs and snRNAs that otherwise fulfilled the “high confidence” criteria were also categorized as “potential”). Candidates were categorized as “confirmed” if they met all of the criteria of the “high confidence” candidates, and in addition to both having a predicted star sequence that was detected in our deep sequencing pool and forming a hairpin with a stable 5′ end and a variable 3′ end.

### Reverse Transcription (RT) of Mature MicroRNA from Total RNA

Total RNA isolated from OVTOKO and HEK293 cell lines was reverse transcribed using the TaqMan® MicroRNA Reverse Transcription Kit from Applied Biosystems (Part Number 4366596) following the manufacturers suggested protocol and specific RT stem-loop primers for mature novel_hsa-bcm-miR-15 sequence and for U6 for an internal control.

### qPCR of cDNA Products

PCR products were amplified from each cDNA sample using the TaqMan MicroRNA Assay together with the TaqMan® Universal PCR Master Mix. Following the manufacturer's recommended reaction conditions, using the Applied Biosystems Veriti system. After 40 cycles of the recommended cycle conditions, data was collected from the machine and analyzed using the Applied Biosystem qPCR software. Using the comparative △△CT method, we used the HEK293 as the reference sample for OVTOKO levels of expression and small RNA U6 as the endogenous control to normalize the expression levels of the novel_ hsa-bcm-miR-15 target.

## Supporting Information

Supporting Data File S1 The set of 132 putative novel miRNAs with sequence copy counts for each sample.
(0.17 MB XLS)

Supporting Data File S2 The predicted set of RNA hairpin secondary structures for the 132 putative novel miRNAs.
(2.74 MB XLS)
